# Uneven Hydrophilic–Hydrophobic Nanoflowers Enhancing Solar Interface Evaporation: Se-Doped Carbon Loaded with Gradient Distribution of CoSe/Co

**DOI:** 10.3390/ma18102409

**Published:** 2025-05-21

**Authors:** Linhui Jia, Zhenhao Liu, Hongxun Hao, Zhongxin Liu

**Affiliations:** 1 School of Chemistry and Chemical Engineering, Hainan University, Haikou 570228, China; zhliu@hainanu.edu.cn; 2 National Engineering Research Center of Industrial Crystallization Technology, School of Chemical Engineering and Technology, Tianjin University, Tianjin 300072, China; hongxunhao@tju.edu.cn

**Keywords:** solar interface evaporation, water cluster evaporation, semiconductor materials, carbon materials, nanoflowers

## Abstract

Solar interface evaporation is a promising technology for sustainable freshwater acquisition. Regulating the hydrophilicity/hydrophobicity of the evaporator can optimize the water transport, heat transfer, and evaporation enthalpy during the evaporation process, thereby significantly improving the evaporation performance. The CoSe/Co-SeC nanoflower was prepared by high-temperature selenization of ZIF-67. Each petal of the nanoflower is loaded with a density-gradient distribution CoSe/Co, forming an uneven hydrophilic and hydrophobic surface that transitions from bottom hydrophilicity to top hydrophobicity. During the evaporation process, the hydrophilic bottom of the petals promotes rapid water supply, while the hydrophobic top of the petals protrudes from the water surface to form a large number of solid–liquid–gas three-phase interfaces. Therefore, water clusters activated by the strong hydrophilic sites at the bottom of the petals can reach the gas–liquid interface after a very short transmission distance and achieve water cluster evaporation. In addition, the nanoflower optimized the heat transfer at the solid–liquid interface and further promoted the increase in evaporation rate through micro-meniscus evaporation (MME). As a result, the evaporation rate and energy efficiency of the CoSe/Co-SeC evaporator are as high as 2.44 kg m^−2^ h^−1^ and 95.5%. This work passes controllable preparation of the gradient CoSe/Co-SeC and shows the enormous potential of micro-hydrophobic and hydrophilic regulation for improving solar interface evaporation performance.

## 1. Introduction

Solar interface evaporation technology (SIE) realizes the efficient absorption of solar energy by combining the evaporation interface and the photothermal conversion interface [1,2,3]. Photothermal materials that can fully absorb sunlight and conduct efficient photothermal conversion at full wavelength are the basis of SIE [4]. At present, photothermal materials such as plasmon metals [5,6], semiconductors [7,8], carbon materials [9,10], and polymers [11,12] have been reported for efficient light absorption and conversion to generate heat. Among these materials, semiconductors are considered promising photothermal conversion materials due to their low infrared radiation, stable physical and chemical properties, good cost-effectiveness, and a wide variety [13,14].

At present, various types of semiconductor materials, such as Ta_2_O_5_ [15], CuS [16], TiO_x_ [17], MoO_x_ [18], and MoS_2_ [19], have been used in SIE. However, the light-absorption capacity of traditional semiconductor materials in the near-infrared region, which accounts for 47% of the total solar energy, is generally poor [20]. Compared with the common transition metal oxides and sulfide semiconductor photothermal materials, the smaller electron ionization energy of Se atoms and the abundant d-orbital electrons can promote the charge delocalization of Se compounds, improve the free-carrier concentration, and trigger the localized surface plasmon resonances (LSPRs) effect, thus producing efficient near-infrared light absorption [21]. Therefore, transition metal selenides are a potential novel semiconductor photothermal material. Shir Abramovich [22], combining the theoretical prediction of energy band structure and dielectric properties, pointed out that the anisotropic dielectric property of NiSe activated multiple LSPRs in the optical range and was released by the unique dielectric dispersion properties of NiSe, resulting in efficient LSPR-assisted solar thermal conversion matching with the solar radiation spectrum. Yao [23] adjusted the thickness of two-dimensional SnSe nanosheets deposited on nickel foam by optimizing the number of laser pulses. Due to the tradeoff between the number of photo-excited electrons and the phonon-emission rate, SnSe nanosheets show good photothermal conversion ability. Jia [24] found that the topological surface states of Bi_2_Se_3_ nanoflowers can enhance photothermal conversion in the near-infrared range through the LSPRs of massless Dirac electrons. In addition, it is an easy and essential design strategy to improve the total spectrum absorption of semiconductor materials by combining carbon-based materials with good near-infrared absorption.

The metal–organic framework material is formed by the periodic combination of inorganic centers (such as Fe, Cu, Zn, and Co) and organic linkers, so its structure is highly adjustable and clearly defined [25]. At present, MOFs have been actively used in many applications, such as gas capture/separation, catalysis, energy conversion, biomedical delivery, and chemical sensing [26]. However, due to the strong bond of metal–ligand in MOFs, some common MOFs (Co-ZIF-67, purple [27]; Zr-UIO-66, white [28]; Cr-MIL-100, green [29]; Zn-ZIF-8, white [30]; Mg-MOF-74, yellow [31]) have poor light-absorption capacity. Pyrolysis of MOFs at high temperatures in the O, S, P, and Se atmosphere has become an advanced method for constructing semiconductor/carbon composites [32,33,34]. The obtained MOF derivatives can inherit the morphology, porous structure, and high-density semiconductor nanoparticles of MOF precursors, to a certain extent [35]. However, as far as we know, the solar photothermal conversion and SIE performance of semiconductor/carbon composites derived from MOF have yet to be explored.

SIE is a multi-physical field problem involving water flow and airflow movement, liquid film and interface heat transfer, and gas–liquid phase transition [36,37,38]. Therefore, heat management, water-path design, and evaporation enthalpy optimization significantly affect SIE performance [39]. One-dimensional and 2D waterways have been constructed using thermal insulation supports to isolate the photothermal evaporation interface from bulk water, limiting the heat to the evaporation surface. Compared with the 1D waterway, the 2D waterway can achieve a sufficient water supply, satisfy more rapid water evaporation, and introduce the Magnani effect based on salt-concentration gradients or temperature gradients to improve the ability of the evaporation film to resist salt crystallization [40,41]. In addition to macro-water-route optimization, micro-water management is an advanced strategy to further improve the thermal management capability of SIE. Guo [42], by constructing hydrophobic nanoislands on the hydrophilic hydrogel surface, formed an uneven surface wetting surface, accelerating the escape behavior of water molecules and achieving an ultra-high evaporation rate of 4.0 kg m^−2^ h^−1^. Yu [43], by growing polypyrrole nano-arrays on hydrophilic carbon cloth, builds a hydrophilic–hydrophobic interface and forms micro-meniscus evaporation. Due to the enhanced heat exchange, larger evaporation area, and enhanced Marangoni mass transfer flow, the evaporation enthalpy of 1510 kJ kg^−1^ and the evaporation rate of 2.16 kg m^−2^ h^−1^ are displayed.

Here, we have innovatively designed a solar interface evaporator. The CoSe/Co-SeC nanoflower with an ordered distribution of micro-hydrophilic and hydrophobic regions was prepared by high-temperature selenization of ZIF-67. The CoSe/Co-SeC nanoflowers formed by ZIF-67 pyrolysis exhibit a typical three-dimensional multilevel structure. Its main body is composed of dozens of nanoscale petal subunits radiating and growing in a central symmetrical manner, forming an open topological structure similar to natural flowers. The density of CoSe/Co nanoparticles embedded in carbon matrix petals gradually decreases from the bottom to the top, forming a unique surface transition from bottom hydrophilicity to top hydrophobicity. The hydrophilic bottom of the petals accelerates water supply, while the hydrophobic top of the petals expands the solid–liquid gas three-phase interface. The water clusters activated by the water level point at the bottom of the petals can be quickly transported to the gas–liquid interface, achieving evaporation of the water clusters and significantly reducing the enthalpy of evaporation. In addition, the optimized heat-conduction distribution of nanoflowers further enhances the evaporation rate through micro-crescent evaporation. As a result, under one sun, the constructed evaporator shows a high evaporation rate of 2.44 kg m^−2^ h^−1^, energy efficiency of 95.5%, and long-term stable and rapid evaporation in salt water.

## 2. Materials and Methods

### 2.1. CoSe/Co-SeC Nanoflowers

Synthesis of ZIF-67: Add 25 mL of 20 mmol 2-methylimidazole methanol solution into 25 mL of 5 mmol Co(NO_3_)_2_·6H_2_O methanol solution to prepare ZIF-67 precursor solution. After that, keep stirring for 30 min. And stand at room temperature for 24 h. The product was washed with ethanol several times and then dried at 60 °C overnight to obtain ZIF-67.

Synthesis of CoSe/Co-SeC: Heat treat ZIF-67 with selenium powder in a tube furnace. Put 200 mg of Se powder in the porcelain boat and place it upstream of the Ar flow, and put 100 mg ZIF-67 in the porcelain boat and place it downstream of the Ar flow. First, heat ZIF-67 and Se powder to 700 °C at a heating rate of 10 °C min^−1^ and keep ZIF-67 and Se powder at 700 °C for 2 h. After that, heat ZIF-67 and Se powder to 1000 °C at a heating rate of 10 °C min^−1^ and keep ZIF-67 and Se powder at 1000 °C for 2 h to ensure the removal of excessive Se powder. The final product is expressed as CoSe/Co-SeC.

Synthesis of Co-GC: Co-GC was obtained by heating ZIF-67 using the same heating program without adding selenium powder.

### 2.2. Methods

Solar vapor-production experiments: The performance of solar vapor generation under the simulated sunlight generated by solar simulator (CEL-HXF300, CEAULIGHT, Beijing, China). The radiation intensity of each test was calibrated by a CEL NP2000 optical power meter (CEAULIGHT, Beijing, China). CoSe/Co-SeC-M was cut into slices of size 2 cm × 2 cm, and water was continuously transported from the container to CoSe/Co-SeC-M using a 3D-printed frame-supported nonwoven fabric. The change in water mass was recorded in real time on a high-precision balance (FA 2004N, JINGHAI, Shanghai, China). The solar evaporation performance of bulk water is obtained by recording the mass change of pure water in the container under sunlight without an evaporator. The solar evaporation performance of bulk water is obtained by recording the mass change of pure water in the container under sunlight without an evaporator. All experiments were performed at an ambient temperature of 28 °C and a humidity of 40% without airflow.

Characterization: FESEM (Verios G4 UC, Thermo scientific, Waltham, MA, USA) and HRTEM (Talos F200X G2, Thermo scientific, Waltham, MA, USA) characterizations were performed to observe the microstructure and morphology of the materials. The absorbance of the samples was studied using a UV-vis-NIR spectrometer (PerkinElmer, Lambda 750s, Waltham, MA, USA) equipped with an integrating sphere. Temperature changes of the samples were recorded on a FLIR A655sc infrared camera. Ion concentrations of water before and after desalination were analyzed by inductively coupled plasma spectroscopy (ICP-MS, Agilent 700, Agilent, Santa Clara, CA, USA). The composition of the samples was analyzed on an X-ray diffractometer (Rigaku Smart Lab, Tokyo, Japan) using Cu Kα radiation (λ = 1.5418 Å). The hydrogen bond composition of water was analyzed using a laser Raman spectrometer (Reflex in Via, Renishaw, Gloucestershire, UK). Valence composition of surface elements was analyzed on X-ray photoelectron spectroscopy (Axis Supra, Kyoto, Japan).

## 3. Results and Discussion

**Figure 1:**
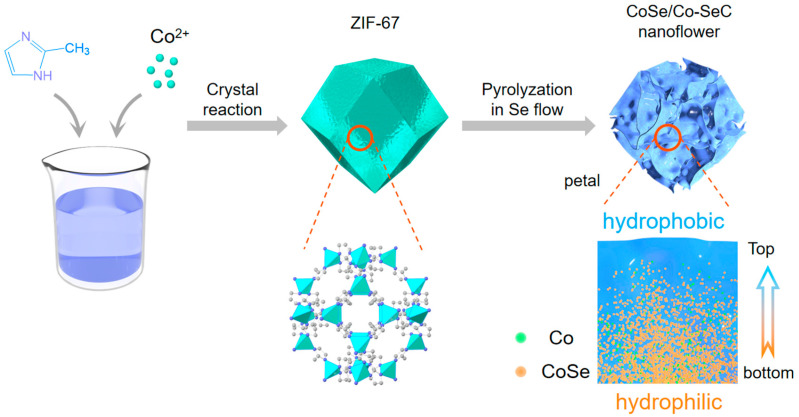
Schematic diagram of the synthesis of CoSe/Co-SeC gradient nanoflowers.

**Figure 2:**
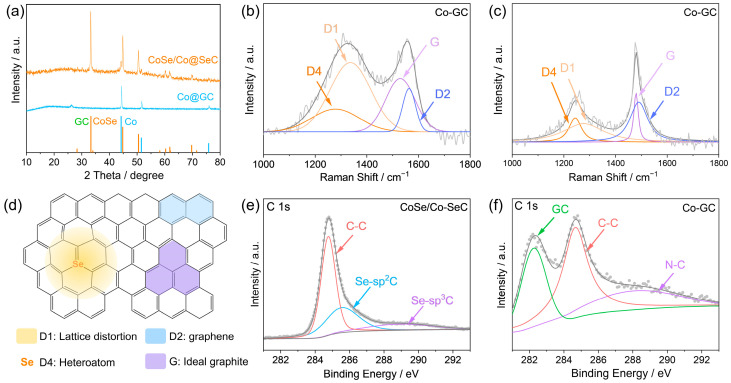
(**a**) XRD pattern of CoSe/Co-SeC and Co-GC. (**b**,**c**) Raman spectra of CoSe/Co-SeC and Co-GC. (**d**) Structure diagram of different kinds of carbon caused by Se doping. (**e**) C 1s high-resolution XPS spectrum of CoSe/Co-SeC. (**f**) C 1s high-resolution XPS spectrum of Co-GC.

ZIF-67 was synthesized according to the method in reference [27]. CoSe/Co-SeC nanoflower photothermal material was prepared by heat treatment of ZIF-67 (Figure 1). The XRD pattern confirmed the successful synthesis of a highly ordered ZIF-67 (Figure S1). As shown in Figure 2a, the XRD results reveal that the material prepared by the high-temperature selenization of ZIF-67 is composed of CoSe and metallic Co. CoSe comes from the decomposition of CoSe_2_, which is the reaction product of Co ions from ZIF-67 and high-temperature Se vapor [44]. The high-temperature environment (1000 °C) facilitates the carbon thermal reduction process, wherein carbon acts as a reductant to partially reduce CoSe, leading to the formation of metallic cobalt (Co) species. No diffraction peak of Se and an obvious carbon peak were observed, possibly due to the formation of an amorphous carbon skeleton by Se doping. However, the synthesized product (Co-GC) without selenium powder showed distinct diffraction peaks of metal Co and a diffraction peak belonging to the ordered carbon structure (Figure 2a). The total proportion of D1 and D4 carbons mainly related to N doping in Co-GC is 55.99% (Table S1).

CoSe, Co, and Se-doped carbon was also confirmed in Raman spectra. As shown in Figure S2, the peaks at 145 cm^−1^, 476 cm^−1,^ and 632 cm^−1^ belong to the A_g_, F_2g,_ and A_1g_ modes of CoSe, which again confirmed the successful synthesis of high-purity CoSe [45]. The peak at 423 cm^−1^ belongs to the E_g_ mode of metal Co [46]. The peak at 769 cm^−1^ belongs to the B_1g_ mode tensile vibration of the Se-C bond [47]. The two strong peaks at 1500 cm^−1^ and 1300 cm^−1^ belong to the G band of the vibration mode of sp^2^ graphite carbon and the D band of amorphous carbon related to defects, respectively. As shown in Figure 2b,c, to determine the structure of carbon nanoflowers, the Raman spectrum of carbon is deconvoluted into five bands (D1, D2, D4, and G) [48]. The G band is the ideal graphite carbon lattice with the E_2g_ symmetry [49], the D1 band is attributed to the lattice distortion of graphite carbon [50], and the D2 band belongs to the monolayer carbon (or graphene carbon) on the surface of graphite crystal [51], which can be regarded as an indicator of the surface area and volume ratio of carbon materials, and the D4 band is related to the existence of heteroatoms in amorphous carbon (Figure 2d) [52,53]. The carbon structure of the CoSe/Co-SeC is shown in detail by counting the area of each fitting peak. The fitting results are summarized in Table S1. The total proportion of D1 and D4 carbon is mainly related to Se doping, which reaches 64.88%. Differently, the total proportion of D1 and D4 carbons related to N doping in Co-GC is relatively low (52.97%). By producing lattice distortion, gap defects, and Se-C structure, highly selenide carbon can generate closely spaced π electron energy levels, accelerating the electron–phonon coupling process and thus strengthening the broadband optical absorption and photothermal conversion capacity [54].

X-ray photoelectron spectroscopy (XPS) was used to analyze the surface-element valence composition of CoSe/Co-SeC. As shown in Figure S3a, the XPS spectrum of CoSe/Co-SeC illustrates the existence of C 1s (284.08 eV), Se 3d (58.08 eV), Co 2p (780.08 eV), and O (530.08 eV). In the high-resolution XPS spectrum of Co 2p, the peaks at 781.2 eV and 797.0 eV belong to the spin-orbital properties of Co 2p_3/2_ and Co 2p_1/2_ (Figure S3b). The energy difference of ∆E = 15.8 eV between the two peaks is close to the value of CoO (∆E = 15.5 ± 0.3 eV), indicating the existence of Co species in the form of Co^2+^ ions. The peaks at 786.1 eV and 803.5 eV are satellite peaks of Co 2p. The existence of two satellite peaks indicates that the electronic states of Co^2+^ ions are in a high-spin arrangement. The sharp peak at 778.5 eV is attributed to the metal Co produced by thermal reduction at high temperatures, which is consistent with the XRD results. According to the literature reports, CoSe’s unique energy band structure and giant electromagnetic anisotropy can cause multiple surface plasmon resonances, thus providing broadband absorption [22]. Additionally, Co nanoparticles can further enhance photothermal conversion performance through its excellent LSPR effect and propagate heat along carbon nanoflowers through phonon–phonon coupling [55,56]. In Figure S3c, the peak at 59.5 eV belongs to Co 3p_3/2_, and the peak at 60.7 eV belongs to Co 3p_1/2_. The peaks of the Se 3d spectrum at 54.3eV, 55.4eV, and 56.5eV belong to Se-Co, Se-sp^2^C, and Se-sp^3^C, respectively (Figure S3c). As shown in Figure 2e, the high-resolution C 1s XPS spectrum can be deconvoluted into three peaks, corresponding to the sp^2^ carbon C-C bond (284.8 eV), Se-sp^2^C bond (285.6 eV), and Se-sp^3^C bond (288.1 eV). The detailed fitting result shows that the ratio of the Se-C bond to the C-C bond in CoSe/Co-SeC is 1.12. The carbon skeleton absorbs incident photons through π–π* transitions, and the structural defects caused by high doping of selenium act as recombination centers to promote non-radiative relaxation of excited electrons, thereby enhancing photothermal conversion [56,57]. On the other hand, the C 1s XPS spectrum of Co-GC shows obvious graphitized carbon peaks, and the degree of defects is low (Figure 2f).

**Figure 3:**
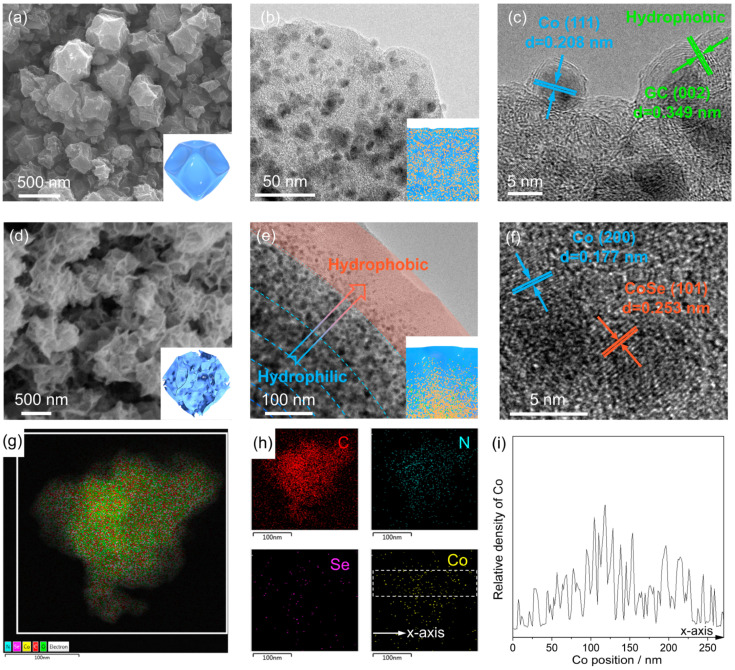
(**a**) The FESEM image of Co-GC; the illustration shows the typical shape of Co-GC. (**b**,**c**) HRTEM image of Co-GC; the illustration shows the disordered distribution of Co nanoparticles on a carbon substrate. The illustration shows the distribution of CoSe nanoparticles (orange) and Co nanoparticles (green) on carbon (blue). (**d**) FESEM image of CoSe/Co-SeC; the illustration shows the typical shape of a single CoSe/Co-SeC nanoflower in a nanoflower aggregate. (**e**,**f**) HRTEM image of CoSe/Co-SeC; the illustration shows the CoSe/Co gradient distribution on nanoflower petals. The illustration shows the distribution of CoSe nanoparticles (orange) and Co nanoparticles (green) on carbon (blue). (**g**,**h**) EDX element-mapping images of CoSe/Co-SeC nanoflowers. (**i**) Statistical analysis results of the density distribution of Co elements along the arrow direction in the selected area (white-dashed box) of Co element in (**h**) (x-axis: position from 0 to 270 nm; solid line: relative density of Co).

Field emission scanning electron microscopy (FESEM) confirmed that the synthesized ZIF-67 exhibited clear edges, angles, and rhombus planes, displaying the classical regular dodecahedron morphology (Figure S4a). The particle-size distribution (1.14 ± 0.52 μm) of ZIF-67 in Figure S4b was quantified (Figure S5a). The wide particle-size distribution originates from the competitive kinetics of nucleation and growth in methanol systems, where higher solvent polarity accelerates crystal aggregation. The FESEM results of Co-GC showed that pyrolysis caused a slight collapse of the ZIF-67 structure (Figure 3a). High-resolution transmission electron microscopy (HRTEM) showed that Co nanoparticles anchored on Co-GC exhibited a disordered distribution (Figure 3b,c), with Co nanoparticles wrapped in graphitized carbon layers at the edges, indicating that the Co-GC surface has strong hydrophobicity. Differently, the pyrolysis in Se vapor resulted in significant collapse of the ZIF-67 structure, forming a nanoflower structure composed of many interwoven protruding petals (Figure 3d). The particle-size distribution of CoSe/Co-SeC nanoflowers is 0.84 ± 0.29 μm (Figure S5b), and, in addition, the nanoflowers are interconnected to form larger aggregates (Figure S4c). The hierarchical nanoflower structure (Figure 3d) promotes multiple reflections of incident light between staggered carbon petals. This geometric light confinement will effectively extend the optical path length, thereby helping to improve the CoSe/Co-SeC light-absorption rate. The HRTEM images provide more detailed structural information of CoSe/Co-SeC nanoflowers. As shown in Figure 3e, amorphous carbon petal nanosheets are anchored with many CoSe/Co nanoparticles, and the particle density presents a decreasing gradient distribution from the inside to the outside of the nanoflower. The metal Co and narrow band gap CoSe with high thermal conductivity can reduce the thermal resistance inside the nanoflower and increase the heat-transfer area. Many dangling bonds and unsaturated ions on the surface of CoSe will significantly increase the hydrophilicity of the high-density CoSe region. Considering the inherent hydrophobicity of pyrolytic carbon, it can be confirmed that an uneven hydrophilic surface structure has formed on the petals.

The polarized surface covered by high-density CoSe at the bottom of the nanoflower will have a strong interaction with water, thus improving the surface wettability and promoting microscopic water transport [58]. The excellent combination of the evaporation interface and photothermal interface helps reduce the heat-transfer loss to bulk water. The HRTEM image in Figure 3f shows that two clear crystal plane spacings of 1.77 Å and 2.53 Å are observed, which correspond to the (101) plane of CoSe and the (200) plane of metal Co, respectively. As shown in Figure 3g,h, the energy-dispersive X-ray (EDX) results showed that the distribution density of CoSe/Co in the nanoflowers exhibited a gradient distribution decreasing from the inside out (Figure 3i).

**Figure 4:**
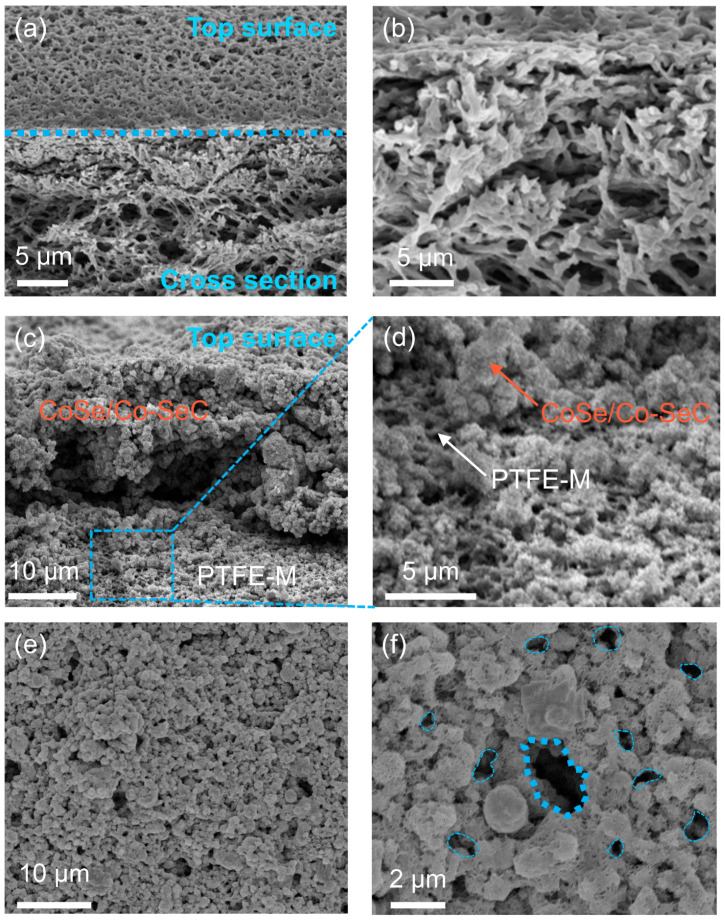
(**a**,**b**) FESEM images of PTFE-M. (**c**,**d**) Angular view of cross section of CoSe/Co-SeC-M. (**e**,**f**) The top surface FESEM image of CoSe/Co-SeC-M. There are large water transfer channels (blue thick dashed line) and small water transfer channels (blue thin dashed line) on the top surface of CoSe/Co-SeC-M in Figure 4f.

The CoSe/Co-SeC photothermal nanoflowers are dispersed in a deionized water solution, and the PTFE membrane (PTFE-M) is used for vacuum filtration to prepare the evaporation membrane (CoSe/Co-SeC-M). The morphology of PTFE-M and CoSe/Co-SeC-M was characterized by FESEM, and the PTFE-M showed a network microporous structure composed of hydrophilic-treated PTFE fibers (Figure 4a,b). The angle view of the cross-section of CoSe/Co-SeC-M shows that CoSe/Co-SeC nanoflowers aggregate at the top of PTFE-M to form a photothermal evaporation layer (Figure 4c). In addition, the vacuum filtration process promotes the deep penetration of nanoflowers into the fiber network structure of PTFE, and this functional gradient interface helps to avoid excessive water supply by the PTFE network (Figure 4d). CoSe/Co-SeC nanoflower stacking forms 0.5–1 μm gap pores and 5–10 μm through-holes (Figure 4e,f). The layered pores are interconnected in the evaporation film, and the water can be quickly pumped through smooth, large channels and transported to each photothermal evaporation interface through numerous branch waterways, ensuring sufficient water replenishment and reducing heat conduction loss to the bottom water.

**Figure 5:**
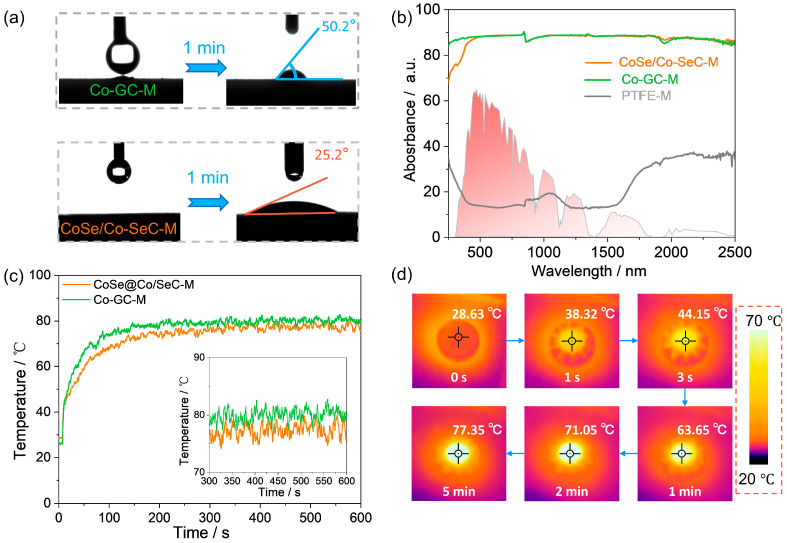
(**a**) Co-GC-M and CoSe/Co-SeC-M wettability test results. (**b**) UV-vis-NIR spectrum of PTFE-M, Co-GC-M and CoSe/Co-SeC-M, and normalized spectral solar irradiation density of air mass 1.5 (AM 1.5 G). (**c**) The temperature-rise curve of CoSe/Co-SeC-M and Co-GC-M under one sun. (**d**) Infrared image of temperature rise of CoSe/Co-SeC-M under 1 sun.

Good thermal, optical, and wetting properties can improve the composite degree of the photothermal interface and evaporation interface to achieve good thermal utilization. The wettability of the PTFE-M and CoSe/Co-SeC-M is characterized by the water-contact angle. The initial PTFE-M can completely absorb the water droplet in 12.7 s (Figure S6). The contact angle of Co-GC-M (evaporation membrane prepared using Co-GC with the same filtration method) remained above 50.2° within 1 min, indicating that graphitized carbon resulted in strong hydrophobicity on the surface of Co-GC (Figure 5a). Furthermore, the water-contact angle of CoSe/Co-SeC-M remains at about 25.2° within 1 min, which is because the inherent hydrophobicity of the SeC skeleton and the hydrophilic CoSe combine to form an uneven hydrophilic structure (Figure 5a). Maintaining a certain wettability is conducive to water infiltration and rapid water transport during evaporation. An ideal photothermal material should have a high-intensity light-absorption performance in the full range of sunlight. The light-absorption performance of PTFE-M, Co-GC-M, and CoSe/Co-SeC-M was studied by UV-vis-NIR (Figure 5b). Total solar absorption (α) based on the solar radiation spectrum can be calculated by the following equation:

(1) $$\alpha =\frac{\int_{300\;nm}^{2.5\;\mu m}\alpha \left(\lambda \right)*A(\lambda ))d\lambda }{\int_{300\;nm}^{2.5\;\mu m}A\left(\lambda \right)d\lambda }$$

In which α(λ) is the solar absorption of evaporation membranes at wavelength λ, and A(λ) is the solar spectral irradiance related to wavelength (AM 1.5G). Due to the numerous optical microcavities of nanoflowers, the additional energy levels introduced by high Se doping in carbon materials and CoSe/Co-efficient LSPRs, CoSe/Co-SeC-M shows a broad optical absorption of up to 88.0% at 250–2500 nm, which is much higher than that of PTFE -M (15.8%). The consistent light absorption of Co-GC-M (88.9%) facilitates a more intuitive comparison of evaporation performance. As shown in Figure 5c,d, the infrared camera records the surface temperature of CoSe/Co-SeC-M, which can rapidly rise to 63.65 °C within 1 min and 77.35 °C after 5 min, which shows that CoSe/Co-SeC has excellent conversion ability from solar energy to thermal energy. Co-GC-M (~80 °C after 5 min) exhibits similar photothermal conversion performance (Figure 5c).

**Figure 6:**
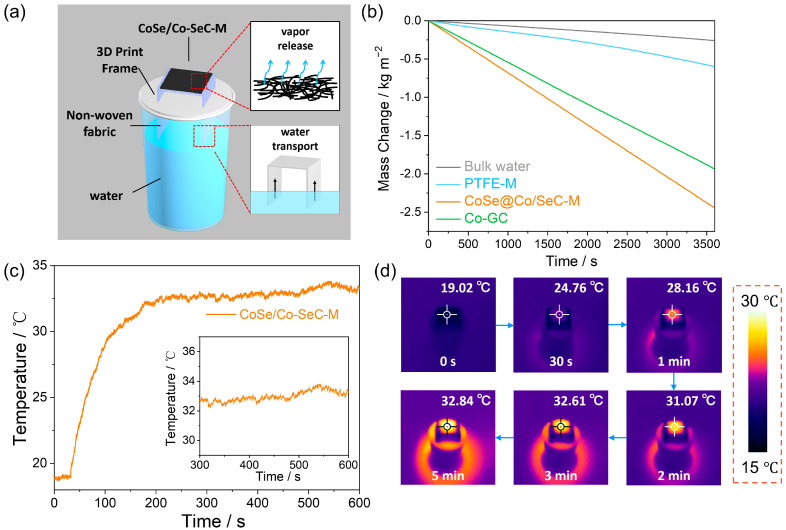
SIE performance of CoSe/Co-SeC-M. (**a**) Schematic diagram of SIE measurement device. (**b**) The mass change of CoSe/Co-SeC-M, Co-GC-M, PTFE-M, and bulk water under one sun. (**c**,**d**) The change of surface temperature of CoSe/Co-SeC-M during evaporation under one sun and the corresponding infrared images.

Figure 6a illustrates the SIE measurement device of CoSe/Co-SeC-M. The evaporation membranes were cut into square pieces of 2 cm × 2 cm size, and 3D-printed frame support nonwoven fabric was used to construct bidirectional 2D water channels for transporting water from the bulk water to the CoSe/Co-SeC-M. The SIE performance test system uses a solar simulator to generate sunlight and records the mass change of water in the evaporation process through a high-precision electronic balance. Under one sun, the time-dependent water-quality change is plotted in Figure 6b. CoSe/Co-SeC-M shows an evaporation rate of 2.44 kg m^−2^ h^−1^, higher than that of Co-GC-M (1.93 kg m^−2^ h^−1^), PTFE-M (0.6 kg m^−2^ h^−1^), and bulk water (0.26 kg m^−2^ h^−1^). During the evaporation process, the infrared thermal imager records the surface temperature change of the CoSe/Co-SeC-M (Figure 6c). The equilibrium surface temperature during evaporation of the CoSe/Co-SeC-M is 32.8 °C, which indicates that the 2D water path has sufficient water supply for the evaporation film and rapid water evaporation. However, the hydrophobic of Co-GC-M obtained from carbonization limits the water supply during solar evaporation, and the equilibrium evaporation temperature of Co-GC-M (40 °C) is relatively high (Figure S8). The corresponding infrared image is shown in Figure 6d. The dark evaporation experiment is used to calculate the equivalent evaporation enthalpy (E_equ_) of water in the CoSe/Co-SeC-M:

(2) $$E_{equ}m_{1}=E_{w}m_{w}$$

where m_1_ is the water mass change in the dark evaporation process of CoSe/Co-SeC-M, and E_w_ and m_w_ are the evaporation enthalpy and the mass change of PTFE-M in the dark evaporation process. CoSe/Co-SeC-M, Co-GC-M, and bulk water showed dark evaporation rates of 0.23 kg m^−2^ h^−1^, 0.19 kg m^−2^ h^−1^, and 0.14 kg m^−2^ h^−1^, respectively (Figure S7). The equivalent evaporation enthalpy of Co-GC-M and CoSe/Co-SeC-M is 1758.3 J g^−1^ and 1442.4 J g^−1^, respectively.

**Figure 7:**
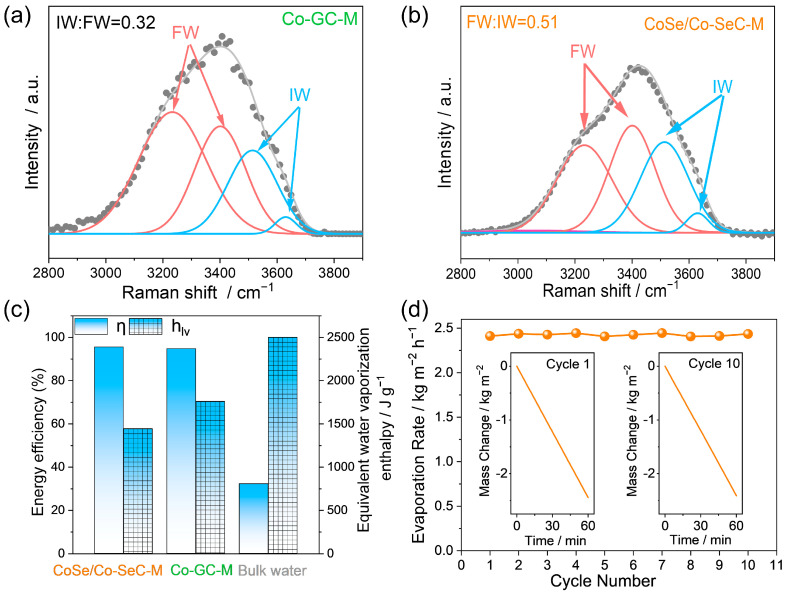
(**a**,**b**) Raman spectra with fitting curves show the ratio of free water and intermediate water in CoSe/Co-SeC-M and Co-GC-M. (**c**) Energy efficiency and evaporation enthalpy of CoSe/Co-SeC-M, Co-GC-M and PTFE-M. (**d**) CoSe/Co-SeC-M ten-cycle stability test, illustration: water mass changes in the first and tenth cycles.

In order to study the reasons for the decrease of evaporation enthalpy, different types of hydrogen-bond-network states of water molecules in the evaporation membranes were studied by Raman spectroscopy. As shown in Figure 7a,b and Figure S9, the Raman spectra of CoSe/Co-SeC-M, Co-GC-M, and PTFE-M can be fitted by four peaks. The peaks at 3230 cm^−1^ and 3400 cm^−1^ correspond to free-water (FW) water molecules with four hydrogen bonds [59]. The peaks at 3514 cm^−1^ and 3627 cm^−1^ correspond to intermediate water (IW) molecules with weak hydrogen bonds or partially broken hydrogen bonds, where the hydrogen bonds of water molecules are partially or completely broken, corresponding to water molecules on the surface of water clusters [60]. The calculated molar ratio of intermediate water (IW) to free water (FW) of CoSe/Co-SeC-M, Co-GC-M, and PTFE-M is 0.51:1 0.32:1, and 0.37:1, respectively, which is attributed to CoSe/Co-SeC-M carbon nanoflower surface with high-density CoSe/Co, attributed to the high-density CoSe/Co induces the formation of water clusters by strongly adsorbing water molecules, thereby reducing the enthalpy of water evaporation. Through the verification experiment of water-cluster evaporation, it was confirmed that CoSe/Co-SeC can promote the evaporation of some water molecules in the form of water clusters (Figure S10).

On the other hand, the uneven hydrophilicity and hydrophobicity of nano flower petals can form numerous individual micro-meniscus evaporation (MME) by rupturing the thick liquid film. Due to the fact that MME brings the hydrophilic zone and gas–liquid interface closer together, water clusters activated by high-density CoSe in the hydrophilic zone can be transferred to the gas–liquid interface through a very short path, thus avoiding the decomposition of the water-cluster-transport process. In addition, MME can also increase the evaporation area, reduce the thickness of the liquid film, and lower the heat-transfer resistance.

**Figure 8:**
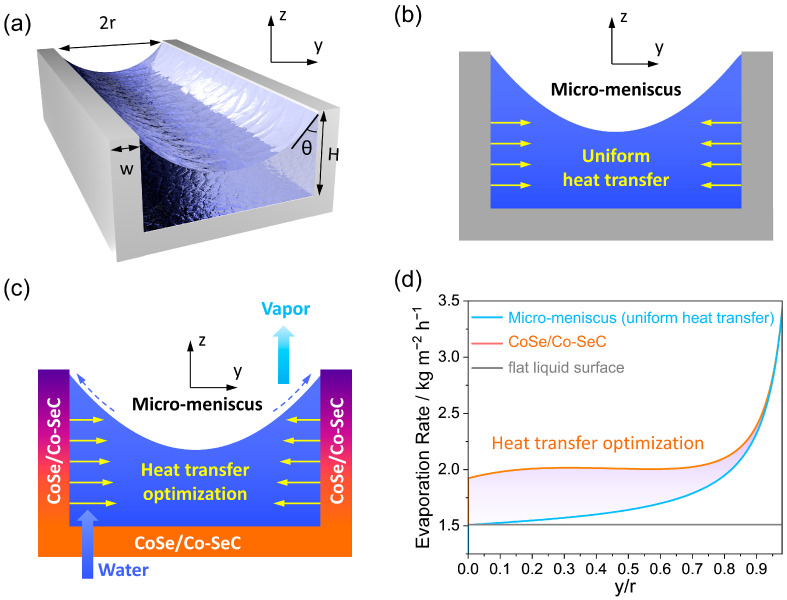
(**a**) Schematic diagram of micro-meniscus evaporation between nanoflower petals. (**b**) The micro-meniscus profile prediction diagram with uniform thermal conductivity. (**c**) The micro-meniscus profile prediction diagram of CoSe/Co-SeC with gradient thermal conductivity. Color coding: purple, hydrophobic carbon region with low thermal conductivity; orange, hydrophilic CoSe/Co region with high thermal conductivity. (**d**) The simulation results of the evaporation-rate distribution of plane thick liquid film, micro-meniscus with uniform thermal conductivity, and gradient CoSe/Co-SeC.

The micro-meniscus profile (Figure S12) between the petals of the CoSe/Co-SeC nanoflowers was determined using the first-order asymptotic solution of the Yang–Laplace equation (Figure 8a) [61]. The local evaporation flux at the liquid–gas interface is determined by the Hertz–Knudsen equation (see calculation method in Supplementary Materials) [62]. The CoSe/Co with strong water affinity can increase the solid–liquid interface bonding strength, reduce the interface temperature saltus step (Figure S13), and improve the interface thermal conductivity, while CoSe/Co also significantly increases the heat-transfer area of the solid–liquid interface. Therefore, compared with the micro-meniscus evaporation with uniform thermal conductivity (Figure 8b), the gradient CoSe/Co-SeC optimized the heat-transfer characteristics in the thick liquid film region (y/r = 0–0.5) (Figure 8c), thus improving the overall evaporation rate of the micro-meniscus (Figure 8d). In addition, the Marangoni flow caused by the temperature gradient in the micro-meniscus accelerates the transfer of water from the bottom of the liquid surface to the top. The solar-evaporative latent energy conversion efficiency of CoSe/Co-SeC-M is calculated by the measured evaporation rate and equivalent evaporation enthalpy:

(3) $$\eta =\frac{mh_{Lv}}{C_{opt}P_{0}}$$

where *m* is the actual evaporation rate, h_Lv_ is the total energy demand of water-phase change, P_0_ is the radiation intensity under one sun, and C_opt_ is the solar radiation concentration. As a result, the energy efficiency of bulk water, Co-GC-M, and CoSe/Co-SeC-M under one sun is 32.19%, 94.75%, and 95.50%, respectively (Figure 7c). In addition, 10 cycles of the solar-evaporation stability at the CoSe/Co-SeC-M interface, the evaporation rate has always been kept above 2.40 kg m^−2^ h^−1^, which shows that the evaporation film has good solar evaporation stability at the interface (Figure 7d). In addition, in the study of SIE stability for 10 cycles under one sun, the evaporation rate of CoSe/Co-SeC-M is always above 2.40 kg m^−2^ h^−1^, which indicates that the evaporation film has good SIE stability.

**Figure 9:**
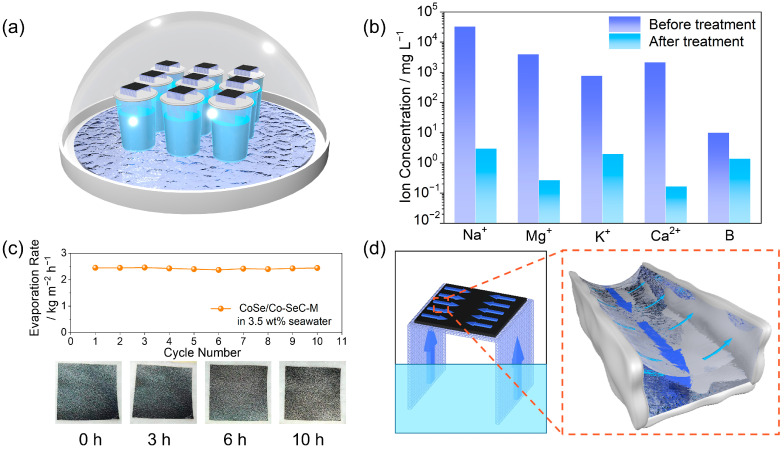
(**a**) Schematic diagram of seawater desalination device of CoSe/Co-SeC-M. (**b**) ICP-MS test results of five main ion concentrations before and after seawater desalination. (**c**) The evaporation rate of CoSe/Co-SeC-M in 3.5 wt% NaCl solution under one sun. The illustration shows the pictures of CoSe/Co-SeC-M salt deposition for 0 h, 3 h, 6 h, and 10 h. (**d**) Schematic diagram of the combination of Marangoni flow of 2D waterway and Marangoni flow of micro-meniscus to promote full-scale water transport. The large arrow in the illustration on the right side of Figure 9d represents the salt-concentration gradient macro-Marangoni flow in the 2D waterway, while the small arrow represents the temperature gradient Marangoni flow in the micro meniscus.

The application potential of CoSe/Co-SeC-M in seawater desalination was evaluated on a self-made seawater desalination device (Figure 9a). As shown in Figure 9b, the concentration changes of five main ions (Na^+^, Mg^2+^, K^+^, Ca^2+^, and B) before and after interface solar desalination were measured by inductively coupled plasma-mass spectrometry (ICP-MS). The concentration of five main ions in the original seawater decreased by three-to-four orders of magnitude, and the overall salt concentration of the freshwater produced was far lower than the safe drinking water standard defined by the World Health Organization (WHO) (<200mg L^−1^). In the long-term seawater desalination process, salt crystallization is one of the main factors that lead to device performance degradation. In the 10 h continuous seawater desalination experiment under one sun, it was found that the evaporation rate of the evaporator remained above 2.37 kg m^−2^ h^−1^, and no salt crystals were observed (Figure 9c). The good salt-cleaning ability of the CoSe/Co-SeC-M device comes from the combination of multi-scale Malangoni flow. As shown in Figure 9d, the salt-concentration gradient macro-Marangoni flow in the 2D waterway promotes the rapid replenishment of bulk water to the high-salinity area of the evaporation film, and the temperature gradient Marangoni flow in the micro-meniscus rapidly transfers the water in the thick liquid film area to the top of the high evaporation rate, avoiding the formation of salt crystals. The surface-tension gradient at the water–air interface generates the interfacial flow from the lower surface-tension region to the higher surface-tension region by causing local viscous stress, thus improving the salt-rejection capability of the evaporator on a full scale. After the cycle-stability test, the CoSe/Co-SeC-M evaporation membrane was washed with pure water to remove salt crystals and surface contaminants. As shown in Figure S14, the regenerated evaporation membrane maintained 97% of its original evaporation rate (2.37 → 2.30 kg m^−2^ h^−1^).

## 4. Conclusions

In summary, through a simple high-temperature selenization strategy, we have achieved a controllable synthesis of gradient CoSe/Co-SeC nanoflower semiconductor/carbon composite photothermal material. Many optical microcavities formed between the petals of a nano-flower improve light capture. The lattice distortion, gap defects, and Se-C structure caused by the high degree of Se-doped carbon skeleton increase the inhomogeneity of the electrostatic potential on the carbon surface, accelerate the electron–phonon coupling process, and promote photothermal conversion. CoSe with anisotropic dielectric properties produces multiple surface plasmon resonances, providing broadband absorption. The density of CoSe/Co on nanoflower petals gradually decreased from inside to outside, resulting in a gradient of hydrophilicity and thermal conductivity. The strong hydrophilic water sites in the bottom hydrophilic region activate water clusters, while the hydrophobic region reduces the transfer distance of water clusters, thereby promoting their evaporation. In addition, the uneven hydrophilic and hydrophobic structure of CoSe/Co-SeC nanoflowers forms a micro-meniscus evaporation mode, which narrows the distance between the hydrophilic water-cluster-activation zone and the gas–liquid interface, shortens the transport path of water clusters from activation to evaporation, and, thus, avoids the decomposition of the water-cluster-transport process. Moreover, the micro-curved liquid surface evaporation mode optimizes the thermal management during the evaporation process, further reducing the enthalpy of evaporation. The composite of multi-scale Marangoni flow comprehensively improves the salt resistance of the evaporation film. As a result, under one sun, the evaporation rate of CoSe/Co-SeC is up to 2.44 kg m^−2^ h^−1^, and the energy efficiency is 95.5%. This work reveals the considerable design potential of semiconductor/carbon materials derived from MOFs to solve the global freshwater shortage problem.

## Data Availability

The original contributions presented in this study are included in the article and Supplementary Materials. Further inquiries can be directed to the corresponding authors.

## Supplementary Materials

The following supporting information can be downloaded at: https://www.mdpi.com/article/10.3390/ma18102409/s1, Figure S1: XRD pattern of ZIF-67; Figure S2: Raman spectra of CoSe/Co-SeC; Figure S3: (a) XPS spectrum of CoSe/Co-SeC. (b) Co 2p high-resolution XPS spectrum of CoSe/Co-SeC. (c) Se 3d and Co 3p high-resolution XPS spectra of CoSe/Co-SeC. (d) Co 2p high-resolution XPS spectrum of Co-GC; Figure S4: (a,b) The FESEM image of ZIF-67; the illustration shows the typical regular dodecahedral shape model of ZIF-67. (c) FESEM image of CoSe/Co-SeC; Figure S5: Particle-size distribution histograms and statistical analysis of ZIF-67 and CoSe/Co-SeC; Figure S6: PTFE-M wettability test results; Figure S7: Dark evaporation rate of CoSe/Co-SeC-M, Co-GC and bulk water; Figure S8: The change of surface temperature of Co-GC-M during evaporation under one sun; Figure S9: Raman spectra with fitting curves show the ratio of free water and intermediate water in PTFE-M; Figure S10: The Li+ concentration in the condensed water obtained by evaporating LiCl solution with pure water and CoSe/Co-SeC-M, the illustration shows a schematic diagram of the water-cluster evaporation verification experiment; Figure S11: Schematic diagram of micro-meniscus evaporation between nanoflower petals; Figure S12: Calculated micro-meniscus profile; Figure S13: Calculated liquid temperature distribution at the micro-meniscus liquid interface; Figure S14: The mass change of regenerated CoSe/Co-SeC-M under one sun; Table S1: Fitting results of carbon Raman spectra in synthesis of CoSe/Co-SeC; Table S2: Evaporation enthalpy of water in bulk water, CoSe/Co-SeC-M and Co-GC.
