# Supplementary material for: Uneven Hydrophilic–Hydrophobic Nanoflowers Enhancing Solar Interface Evaporation: Se-Doped Carbon Loaded with Gradient Distribution of CoSe/Co
Source: Materials (Basel). 2025 May 21;18(10):2409. doi: 10.3390/ma18102409 (PMC12113247; doi:10.3390/ma18102409)
Supplement: Supplementary file 1 [file materials-18-02409-s001.zip › materials-3613977-supplementary.pdf]

Supporting information

Uneven hydrophilic – hydrophobic nanoflowers enhancing solar interface evaporation: Se-doped carbon loaded with gradient distribution of CoSe/Co

Linhui Jia*, Zhenhao Liu, Hongxun Hao*, Zhongxin Liu*

School of Chemistry and Chemical Engineering, Hainan University, Haikou 570228, P. R. China.

*Corresponding authors: Linhui Jia, Zhongxin Liu, Hongxun Hao

E-mail address: jlh@hainanu.edu.cn (LH. Jia), hongxunhao@tju.edu.cn, liuzhongxin@hainanu.edu.cn

Experimental part

Materials and chemicals: $Co(NO_3)_2 \cdot 6H_2O$, 2-methylimidazole and methanol were obtained from Sinopharm Chemical Reagent Co., Ltd. Se powder was obtained from McLean Reagent Co., Ltd. All initial reagents are used directly after purchase without further treatment.

Verification experiment of water-cluster evaporation: In order to investigate the mechanism of CoSe/Co-SeC-reducing enthalpy of evaporation, a water-cluster evaporation verification experiment of CoSe/Co-SeC was conducted. The experiment involves adding LiCl with strong hydration ability to water, and then detecting the Li^+ concentration in the cooling water after CoSe/Co-SeC evaporation using ICP-MS. As shown in **Figure S10**, the Li^+ concentration in the condensate of CoSe/Co-SeC is much higher than that in the condensate produced without CoSe/Co-SeC. This is attributed to CoSe/Co-SeC's ability to induce water molecules to evaporate by forming water clusters composed of several to dozens of water molecules, which then carry Li^+ to the condensed water.

Simulation of micro-meniscus evaporation rate: To study the inhomogeneous hydrophobicity and heat-transfer optimization of the gradient CoSe/Co-SeC, we use the first-order asymptotic solution of the Yang–Laplace equation to study the liquid surface profile between the petals of CoSe/Co-SeC nanoflowers. We define the width-to-space ratio as δ=w/2r, where w is the thickness of nanoflower petals, 2r is the spacing between nanoflower petals, and H is the height of nanoflower petals (**Figure S11**). The contact angle θ=25.2 ° defined the profile function of the liquid–gas interface:

$$z(y) = (1 - \sin\theta)\frac{y^2 - r^2}{r\cos\theta} + O(\delta^3) \tag{S1}$$

The calculated micro-meniscus curve is shown in **Figure S12**. Once the profile of the liquid–air meniscus is determined, the local evaporation flux at the liquid–air interface can be determined by the Hertz–Knudsen equation: [1]

$$\dot{m} = \frac{2\sigma}{2 - \sigma}\sqrt{\frac{M}{2\pi R}}\left(\frac{P^s(T_{lv})}{T_{lv}^{0.5}} - \frac{P_v}{T_v^{0.5}}\right) \tag{S2}$$

Where $\dot{m}$ refers to mass flux and adjustment coefficient σ=0.3, M is the molar

mass of the fluid, R is the molar gas constant, P^s is the saturated vapor pressure of the liquid, T_{lv} is the liquid temperature at the interface, P_v is the vapor pressure at the interface, and T_v is the vapor temperature at the interface. The energy consumption of the liquid–gas interface can be written as:

$$\dot{q} = \dot{m}h_{fg} = k_l(T_s - T_{lv})\left(\frac{\sin\alpha}{r-y} + \frac{\cos\alpha}{H+z(y)}\right) \quad \text{(S3)}$$

k_l is the Knudsen number, while α indicates the turning angle at the gas–liquid interface and is determined by the contour of the meniscus: dz/dy=tan α (**Figure S11**). T_s is the temperature of the solid in contact with the steam. By solving equations (S1) and (S2), the distribution of liquid surface temperature and mass flux on the meniscus is obtained. Combined with infrared data, the T_s of CoSe/Co-SeC is defined as T_s=305.8-20z. The predicted T_{lv} curve is shown in **Figure S13**, and the corresponding mass-loss rate is shown in **Figure 8d**. Compared with the evaporation of curved liquid surface with uniform thermal conductivity (**Figure 8b**), CoSe/Co-SeC optimizes the thermal resistance of the thick liquid film region (y/r=0.0-0.5), thus improving the overall evaporation rate of the micro-meniscus.

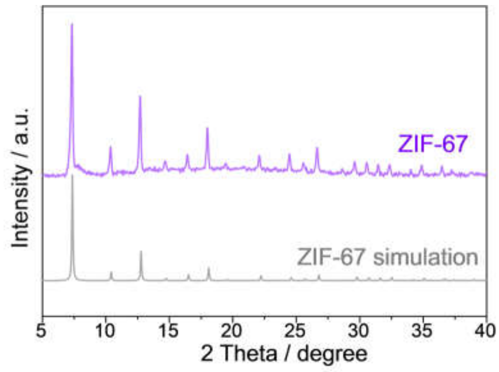

Figure S1. XRD pattern of ZIF-67.

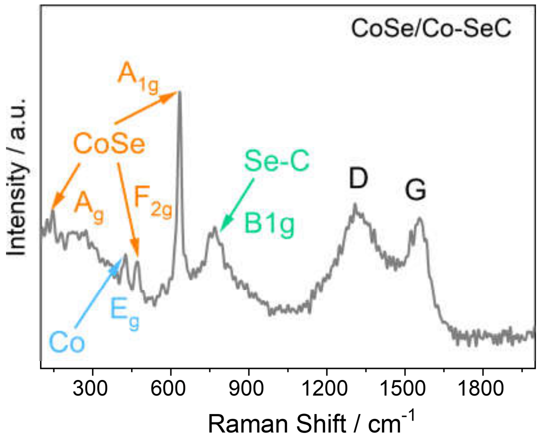

Figure S2. Raman spectra of CoSe/Co-SeC.

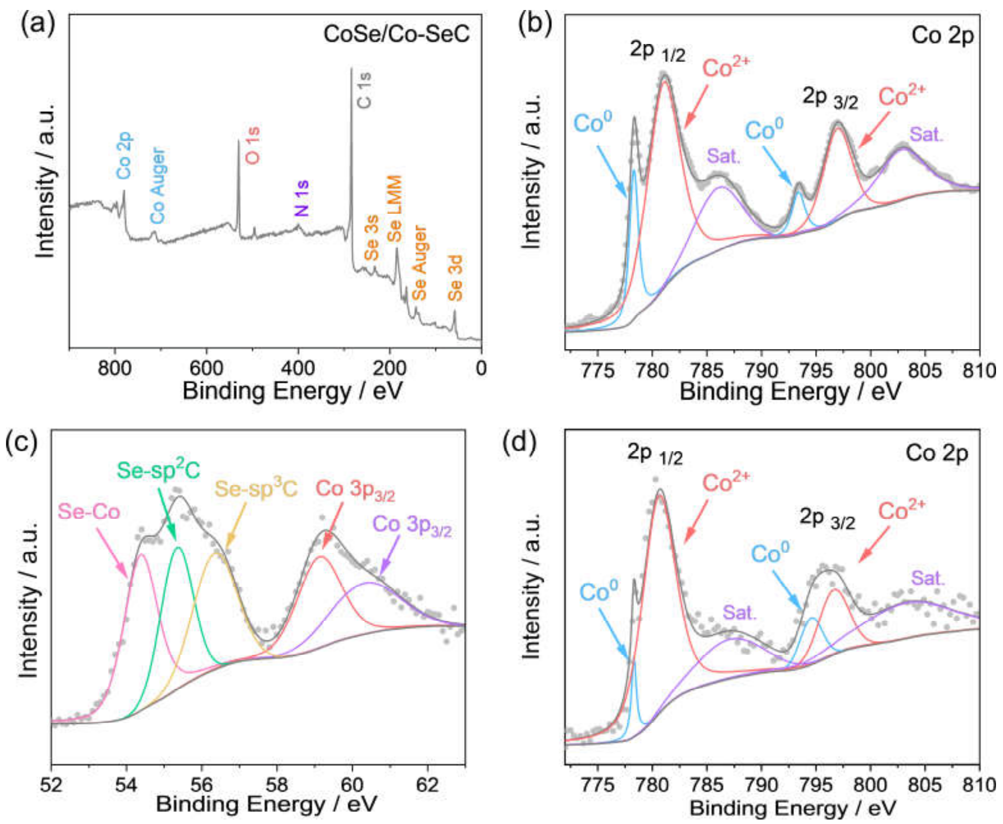

Figure S3. (a) XPS spectrum of CoSe/Co-SeC. (b) Co 2p high-resolution XPS spectrum of CoSe/Co-SeC. (c) Se 3d and Co 3p high-resolution XPS spectra of CoSe/Co-SeC. (d) Co 2p high-resolution XPS spectrum of Co-GC.

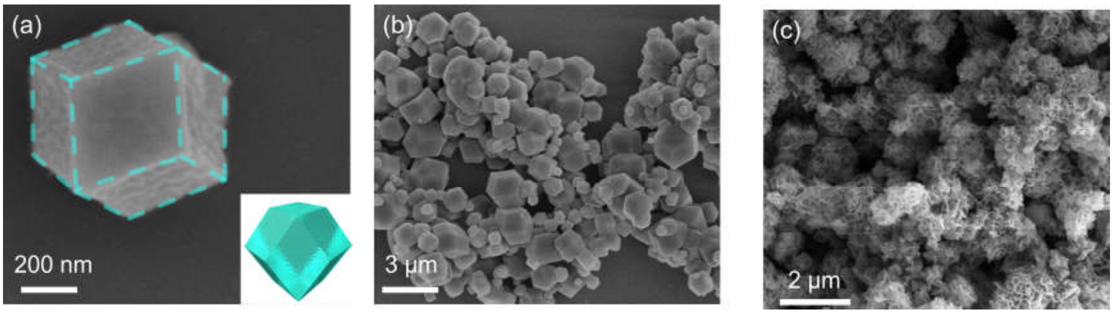

Figure S4. (a,b) The FESEM image of ZIF-67; the illustration shows the typical regular dodecahedral shape model of ZIF-67. (c) FESEM image of CoSe/Co-SeC.

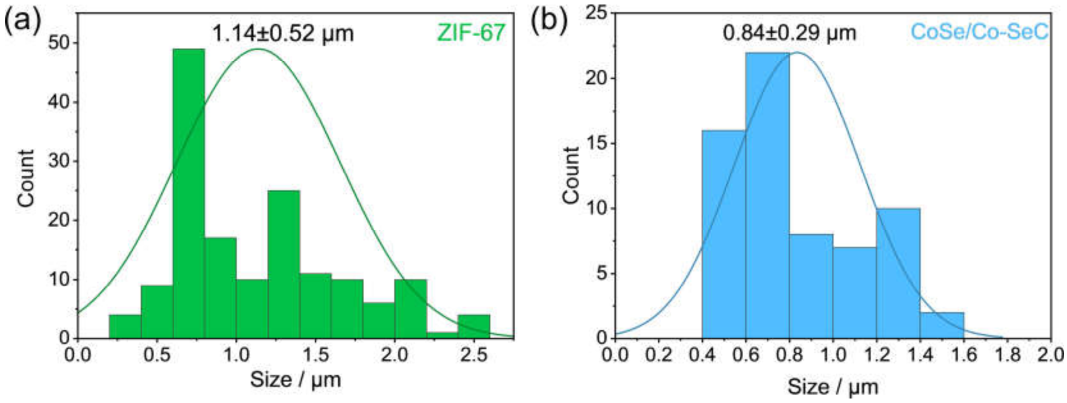

Figure S5. Particle-size distribution histograms and statistical analysis of ZIF-67 and CoSe/Co-SeC.

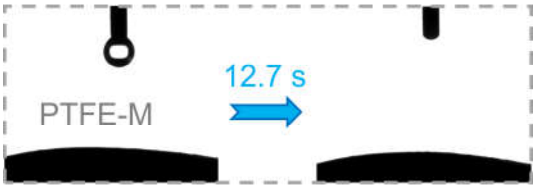

Figure S6. PTFE-M wettability test results.

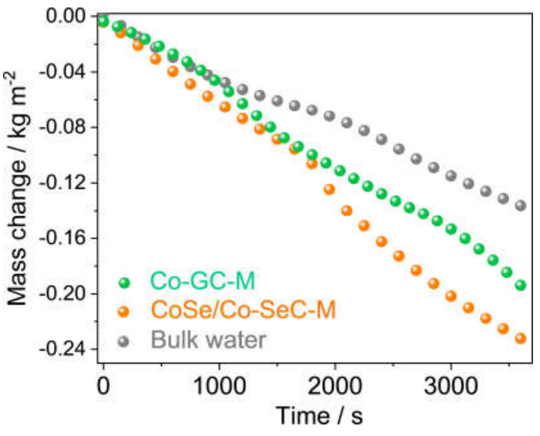

Figure S7. Dark evaporation rate of CoSe/Co-SeC-M, Co-GC and bulk water.

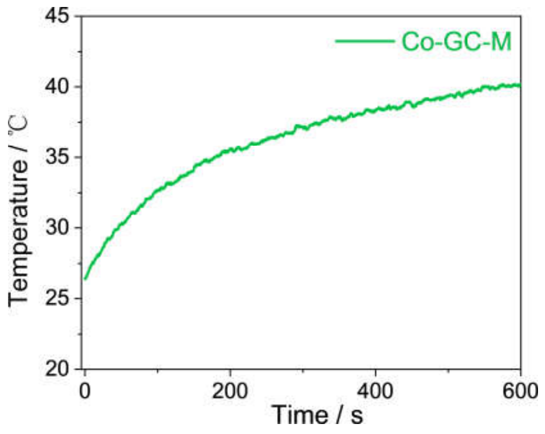

Figure S8. The change of surface temperature of Co-GC-M during evaporation under one sun.

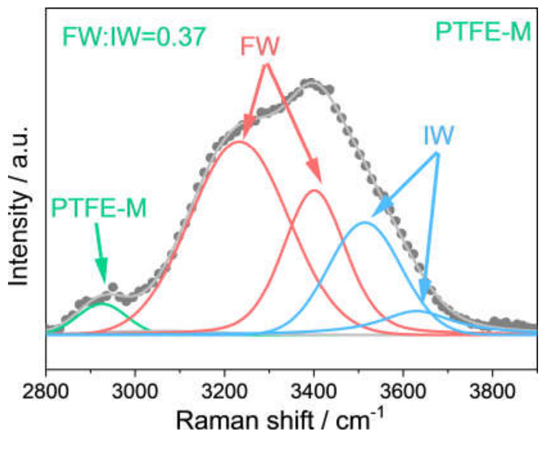

Figure S9. Raman spectra with fitting curves show the ratio of free water and intermediate water in PTFE-M.

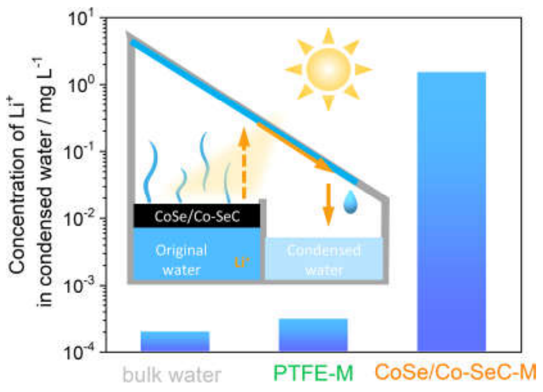

Figure S10. The Li^+ concentration in the condensed water obtained by evaporating LiCl solution with pure water and CoSe/Co-SeC-M, the illustration shows a schematic diagram of the water-cluster evaporation verification experiment.

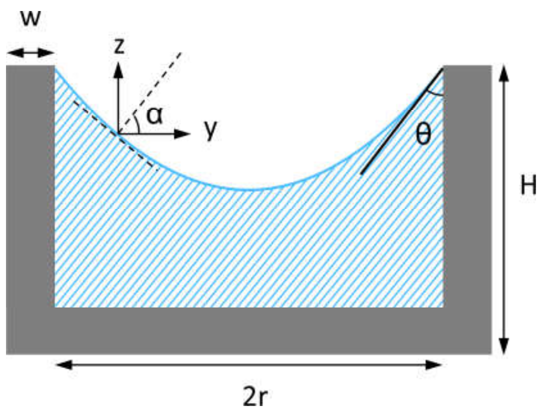

Figure S11. Schematic diagram of micro-meniscus evaporation between nanoflower petals.

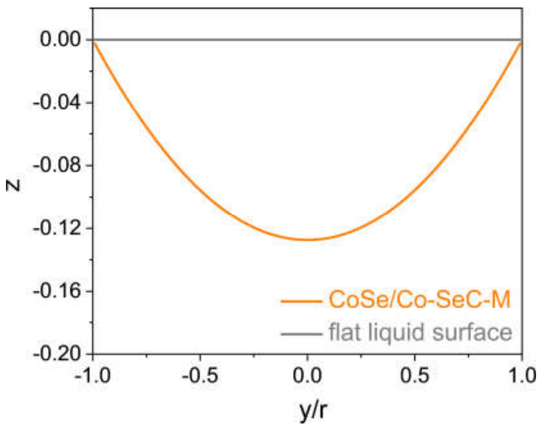

Figure S12. Calculated micro-meniscus profile.

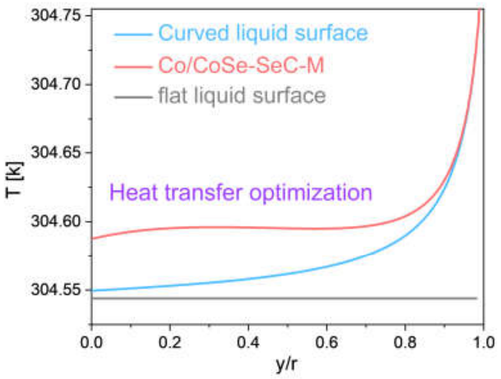

Figure S13. Calculated liquid temperature distribution at the micro-meniscus liquid interface.

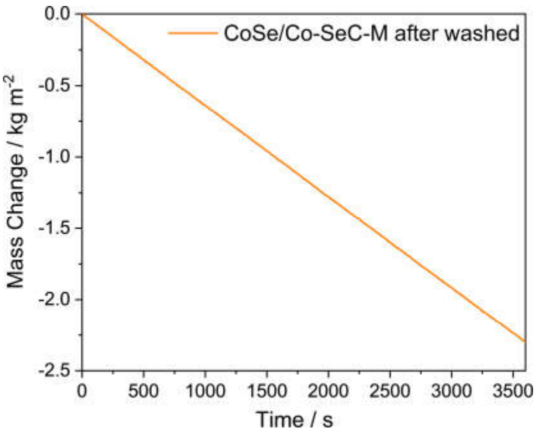

Figure S14. The mass change of regenerated CoSe/Co-SeC-M under one sun.

Table S1. Fitting results of carbon Raman spectra in synthesis of CoSe/Co-SeC

		D4	D1	G	D2
Peak area (%)	CoSe/Co-SeC	17.90	46.98	25.87	9.26
	Co-GC	15.39	37.58	11.21	35.81

Table S2. Evaporation enthalpy of water in bulk water, CoSe/Co-SeC-M and Co-GC.

	Bulk water	CoSe/Co-SeC-M	Co-GC
Mass change (kg m^{-2} h^{-1})	0.14	0.23	1.93
Vaporization enthalpy (J g^{-1})	2500.0	1442.4	1758.3

Note S1. Estimation of water-evaporation enthalpy in CoSe/Co-SeC-M

According to the reported method[2], the evaporation enthalpy of water in CoSe/Co-SeC-M and bulk water was estimated through dark evaporation experiments. In the controlled experiment, bulk water with the same surface area and evaporators of CoSe/Co-SeC-M were placed in a closed container with supersaturated $MgCl_2$ aqueous solution to maintain the relative humidity at about 33%. The experiment was conducted at room temperature (25 °C) and ambient air pressure under dark conditions for 12 hours. Due to the dark conditions, the energy absorbed from the environment is the only energy to maintain water evaporation. Generally, heat transfer is divided into three ways, namely heat conduction, heat convection, and heat radiation. Since the sample/air interface remains static during the test, thermal convection can be ignored. Under dark conditions, the negligible radiant heat transfer from the environment is too weak to heat the water near the interface. Therefore, heat conduction determines heat flow, which can be described by the following equation:

$$Q = \frac{kA(T_2 - T_1)}{d}$$

Where k, A, and d are the thermal conductivity of the heat transfer, the heat-transfer interface area, and the thickness of the heat-transfer layer. T_2 and T_1 refer to the ambient and evaporating surface temperatures, respectively. In our experiment, the temperature between bulk water–air and CoSe/Co-SeC-M–air is almost the same. The ambient medium is air, which means that the temperature gradient above the evaporation surface is similar. In the case of different bulk water or CoSe/Co-SeC-M, k, A, d, T_2, and T_1 may be the same. Therefore, it can be assumed that the energy input from the environment to the test sample is the same. The dark evaporation rate of bulk water and CoSe/Co-SeC-M is shown in **Figure S7**. Therefore, the equivalent evaporation enthalpy (E_{equ}) of water in CoSe/Co-SeC-M can be calculated by the following formula:

$$E_{equ} m_1 = E_w m_w$$

Where m_l is the mass change of water on the CoSe/Co-SeC-M evaporator, E_w, and m_w are the evaporation enthalpy and the mass change of water on bulk water during evaporation. The experimental results are summarized in **Table S2**. The estimated

evaporation enthalpies of water in CoSe/Co-SeC-M are 1442.4 J g^{-1}.

References

[1] Farokhnia N., Irajizad P., Sajadi S. M., Ghasemi H. Rational Micro-Nano Structuring for Thin Film Evaporation [J]. Journal of Physical Chemistry C, 2016: acs.jpcc.6b01362.

[2] Zhao Fei, Zhou Xingyi, Shi Ye, Qian Xin, Alexander Megan, Zhao Xinpeng, Mendez Samantha, Yang Ronggui, Qu Liangti, Yu Guihua. Highly efficient solar vapour generation via hierarchically nanostructured gels [J]. Nature Nanotechnology, 2018, 13(6): 489-+.
